# Genome-Wide Association Study of Coronary Artery Disease

**DOI:** 10.4061/2010/790539

**Published:** 2010-09-21

**Authors:** Naomi Ogawa, Yasushi Imai, Hiroyuki Morita, Ryozo Nagai

**Affiliations:** ^1^Department of Cardiovascular Medicine, Graduate School of Medicine, The University of Tokyo, 7-3-1 Hongo Bunkyo-ku, Tokyo 113-8655, Japan; ^2^Division of Clinical Genome Informatics, Graduate School of Medicine, The University of Tokyo, Japan; ^3^Department of Translational Research for Healthcare and Clinical Science, Graduate School of Medicine, The University of Tokyo, Japan

## Abstract

Coronary artery disease (CAD) is a multifactorial disease with environmental and genetic determinants. The genetic determinants of CAD have previously been explored by the candidate gene approach. Recently, the data from the International HapMap Project and the development of dense genotyping chips have enabled us to perform genome-wide association studies (GWAS) on a large number of subjects without bias towards any particular candidate genes. In 2007, three chip-based GWAS simultaneously revealed the significant association between common variants on chromosome 9p21 and CAD. This association was replicated among other ethnic groups and also in a meta-analysis. Further investigations have detected several other candidate loci associated with CAD. The chip-based GWAS approach has identified novel and unbiased genetic determinants of CAD and these insights provide the important direction to better understand the pathogenesis of CAD and to develop new and improved preventive measures and treatments for CAD.

## 1. Introduction

Coronary artery disease (CAD) including myocardial infarction (MI) is a leading cause of death worldwide [1, 2]. The well-known conventional coronary risk factors include age, male sex, hypertension, diabetes mellitus, hypercholesterolemia, smoking and family history, which have been repeatedly demonstrated in multiple epidemiological studies [3–5]. Lifestyle and environmental factors play an important role in the pathogenesis; however, genetic predisposition is also thought to contribute to CAD/MI since these diseases cluster in families [6].

In the epidemiological studies using twins, the relative hazard of death among men from CAD when one's twin died of CAD before the age of 55 years, as compared with the hazard when one's twin did not die before 55, was 8.1 for monozygotic twins and 3.8 for dizygotic twins [7]. The recent epidemiological survey in the Framingham study showed that parental cardiovascular disease independently predicted future offspring events [8]. In this survey, participants with at least one parent with premature CAD had greater risk for events with age-adjusted odds ratios (ORs) of 2.6 for men and 2.3 for women compared with those with no parental CAD. These results support further research into genetic determinants of CAD risk. Elucidating the genetic determinants would improve risk assessment and provide better measures for prevention and treatment. 

As the molecular biology and genetics had progressed, the genetic backgrounds were explored in the several genes which were thought to contribute to the pathogenesis of atherosclerosis and conventional coronary risk factors. Candidate gene studies tested the hypothesis that proteins known to be involved in the pathogenesis of atherosclerosis carry variants that affect their protein functions and the risk of developing CAD. In 1992, Cambien et al. [9] explored a possible association between CAD and a variation found in the gene encoding angiotensin-converting enzyme (ACE). The polymorphism ACE/insertion/deletion (ACE/ID) is strongly associated with the level of the circulating enzyme. They reported that the deletion homozygote (DD genotype), which was associated with higher levels of circulating ACE, is significantly more frequent in patients with MI than in controls. 

The representative variants associated with CAD/MI found by candidate gene approach are listed in Table 1. However, those candidate genes were not always reproducible in multiple studies later on. One of the reasons for poor reproducibility is that many of the study samples were not large enough with some exceptions to identify disease-associated genetic variants with odds ratio <2.0. In addition, candidate gene studies only tested a single to few variants for association with CAD and these approaches cannot discover unknown novel variants and also cannot evaluate how strong each variant contribute to the susceptibility to CAD. Therefore, the candidate gene approach resulted in only limited success in the elucidation of genetic risks for CAD.

In parallel with candidate gene studies, other strategies were carried out to interrogate the entire human genome without hypotheses on which genes may be responsible for disease risk. One of the strategies is genome-wide linkage analysis and it is based on the Mendelian cosegregation of a genetic marker within a family. However, great efforts had to be made in order to collect sufficient numbers of affected sibling pairs. Only a small number of studies were successfully performed and few genetic loci (2q21-22, Xq23-26 [10], myocyte enhancer factor-2 (*MEF2A*) [11], arachidonate 5-lipoxygenase-activating protein (*ALOX5AP*) [12], leukotriene A4 hydrolase (*LTA4H*) [13]) were detected to be associated with CAD (Table 2). These genes had never been suggested as causative genes before these family-based studies, suggesting the effectiveness of this approach in detection of novel genetic determinants. However, those associations were not always replicated. Such a family-based study has been frequently used to identify new loci in monogenic diseases, but the application of this strategy to multifactorial diseases is relatively limited. 

In this situation, whole genome analysis in a case-control study design had gradually emerged around the beginning of the 21st century. Resources including the single nucleotide polymorphism (SNP) databases, major technological advances in high-throughput genotyping, and methods of data processing and statistical analysis allow researchers to confront limitations in previous approaches. Here, we introduce the representative genome-wide case-control association studies in the next two sections.

## 2. Establishment of J-SNP Database and Whole Genome Approach in Japan

Beginning in 2000, the Prime Minister's Millennium Project (J-SNP) was launched in Japan and about hundred thousand SNPs located in genes or in adjacent regions that might influence the coding sequence of the genes were identified in Japanese population. J-SNP established a web-based database and allowed researchers access to high quality SNP data [14]. Genome-wide association studies using this SNP database were performed in our country in many important clinical fields including cardiovascular diseases, diabetes, renal dysfunction and autoimmune collagen diseases.

As the first genome-wide case-control association study in the world, Ozaki et al. [15] used 92,788 gene-based SNP markers and identified that the homozygosity in two SNPs in lymphotoxin A (*LTA*) at 6p21 was significantly associated with increased risk for MI in Japanese (odds ratio (OR) = 1.78). In vitro analyses showed that one functional SNP (Thr26Asn) caused a twofold increase in induction of several inflammation-related cell-adhesion molecules including vascular cell adhesion molecule 1 (VCAM1) in vascular smooth-muscle cells. Moreover, the SNP located in intron 1 of LTA enhanced the transcriptional level of LTA. These results indicated the variants in the LTA are risk factors for MI and implicated LTA as a novel pathogenic factor for MI. In the same year, LTA knockout mouse was shown to be resistant to atherosclerosis [16]. Double knockout mice of apolipoprotein E (ApoE) and LTA (ApoE−/−LTA−/− mice) showed less extent of atherosclerosis than ApoE−/−LTA+/+ mice, indicating LTA deteriorates atherosclerosis in vivo, consistent with the result of the genetic association study of LTA as a genetic risk for atherosclerotic disease.

Subsequently, they identified SNPs that were significantly associated with MI in lectin, galactoside-binding, soluble, 2 gene (*LGALS2*) [17], proteasome subunit alpha type 6 gene (*PSMA6*) [18], myocardial infarction associated transcript (*MIAT*) [19], inter-alpha (globulin) inhibitor 3 gene (*ITIH3*) [20] and BRCA1-associated protein gene (*BRAP*) [21] mainly by functional approaches. All the six causative genetic regions are related to inflammatory process in vasculature and are thought to contribute to the process in atherosclerotic changes through its inflammatory functions and thus increase the risk of MI. 

## 3. Chip-Based GWAS and Novel Candidate Genetic Determinants for CAD

### 3.1. HapMap Project

Genome-wide association studies based on the J-SNP database (approximately 100,000 SNPs) have detected several SNPs which had significant association with myocardial infarction. However, the J-SNP database does not cover SNPs in intergene regions. In the meantime, the International HapMap Project was conducted to create a public genome-wide database of common SNPs and enable systematic studies of common SNPs for their potential role in human disease [22, 23]. The Project analyzed DNA samples from 90 people with European ancestry, 90 Yoruba people in Nigeria, 44 Japanese and 45 Han Chinese and has now genotyped over 3.1 million SNPs in each of these populations. However, testing all of these SNPs in a person's chromosomes would be extremely expensive. Adjacent SNPs across the genome are correlated each other, a phenomenon known as linkage disequilibrium and SNPs that are inherited together were compiled into “haplotypes”. A haplotype block may contain many SNPs, but only a few “tag” SNPs can provide most of the information on the pattern of genetic variation in the block. The HapMap project identified these “tag” SNPs within haplotypes that uniquely identify these haplotypes. The HapMap data allowed efficient design of Chip-based genome association studies and allowed investigators to genotype far fewer SNPs while still retaining statistical power to find genetic variants related to common illness. 

### 3.2. High throughput SNP Genotyping Platforms

The development of dense genotyping chips enables genotyping up to 1 million SNPs on a single small chip. This chip technology allowed genome-wide association studies (GWAS) to be performed on a large numbers of subjects. Chip-based GWAS typically involves genotyping approximately a few thousands cases with a disease and a few thousands of controls for about 500,000 tag SNPs. Since there are 500,000 comparisons per study, there is a high potential for false positive results. The proposed solution for that is applying the stringent P value using the Bonferroni correction for multiple tests. In that case, *P* value will be 0.05 divided by 500,000 and that is 0.0000001 (10^−7^) and this stringent *P* value is often termed as ‘genome-wide significance'. The most statistically significant variants identified in the initial case-control analysis are tested for replication in subsequent case-control studies. In GWAS method, associations between SNPs and the diseases are made free of bias of particular candidate genes. This makes the possibility of obtaining novel and unbiased information and provides the important direction to better understand the pathophysiology of the disease. For CAD, three chip-based GWAS were simultaneously reported in 2007 and all of them showed the significant association between CAD and SNPs on chromosome 9p21.

### 3.3. 9p21 and other Chromosomal Loci Associated with CAD/MI Detected in Chip-Based GWAS

**Figure 1:**
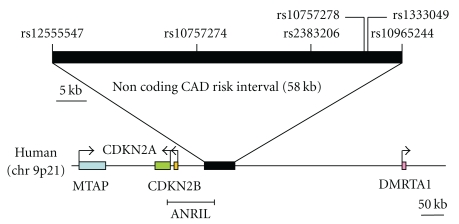
The 58 kb non-coding CAD risk interval on chromosome 9p21.

Helgadottir et al. enrolled a total of 4587 MI cases and 12,767 controls and genotyped total 305953 SNPs using Illumina Hap300chip (Illumina) [24] (Table 3). All the participants were European descent. They identified disease association variant located in 9p21, adjacent to the tumor suppressor genes cyclin-dependent kinase inhibitor 2A (*CDKN2A*) and cyclin-dependent kinase inhibitor 2B (*CDKN2B*) with great statistical significance. This region had never been estimated to be associated with susceptibility to MI. They showed the allele G of the SNP rs10757278 (Figure 1) showed the strongest association with MI. The ORs for heterozygous and homozygous carriers of the risk allele G were 1.26 and 1.64, respectively. The ORs for early-onset MI (MI before the age of 50 for males and before the age of 60 for females) are 1.49 and 2.02 for heterozygous and homozygous carriers of the risk allele, respectively. They estimated the population attributable risk is 21% for MI in general and 31% for early-onset cases. 

The SNPs on chromosome 9p21 associated with MI are located in the same disequilibrium block of the one which contains *CDKN2A* and *CDKN2B*. These genes encode two members of the inhibitors of CDK4 (Ink4) family of cyclin-dependent kinase inhibitors, p16^Ink4a^ and p15^Ink4b^, and a completely unrelated protein called ARF. The p16^Ink4a^ and p15^Ink4b^ which activates retinoblastoma (Rb) family members and ARF which activates p53 were shown to be upregulated in cancer cells. They play a critical role in cell proliferation and aging, senescence and apoptosis [25, 26]. However, sequencing 93 early-onset MI patients across these genes did not reveal obvious causal functional variants or variants that could account for the correlation of rs10757278 to MI. The linkage disequilibrium block also contains two exons of the transcript hypothetical methylthioadenosine phosphorylase fusion protein mRNA, however the functional significance of the variants in this region remains to be elucidated.

McPherson et al. also identified a 58-kilobase region on chromosome 9p21 that was consistently associated with CAD in six independent samples consisted of 4306 cases and 20119 controls from four Caucasian populations [27]. They identified two SNPs rs10757274 and rs2383206 on 9p21 that are significantly associated with incident of CAD. The risk allele was associated with and ~15 to 20% increase risk of CAD in the 50% individuals who are heterozygous and ~30 to 40% increase in the 25% who are homozygous for the allele. 

They further genotyped surrounding region of these SNPs in detail and found that eight additional SNPs at the locus spanning a 58-kb region were significantly associated with CAD. Again, the 58-kb region does not contain any annotated genes. however, the region overlaps a newly annotated noncoding RNA called noncoding RNA in the *INK *locus (*ANRIL*) [28]. *ANRIL* consists of 20 exons subjected to alternative splicing. The whole blood RNA expression levels of short variants of *ANRIL* are increased and the expression levels of the long variant are decreased in subjects homozygous for the risk alleles. There is also a positive correlation between transcript levels of the long variant of *ANRIL* and *CDKN2B* [29]. 

The study using genetically engineered mice showed that the deletion of orthologous 70-kb non-coding interval on mouse chromosome 4 (chr4^△70kb/△70kb^mice) highly reduced cardiac expression of two neighboring genes *CDKN2A* and *CDKN2B*. Primary culture of smooth muscle cells from chr4^△70kb/△70kb^mice showed increased proliferation and diminished senescence, the features relevant to atherosclerosis [30]. These findings indicated the noncoding interval is involved in the disease process via gene-regulatory effects on *CDKN2A* and *CDKN2B*. 

The Wellcome Trust Case Control Consortium (WTCCC) study which enrolled 1926 case subjects with CAD and 2938 controls also reported the powerful association between the SNPs on chromosome 9p21 and CAD [31]. The strongest signal was seen at rs1333049, however the associations were seen for SNPs across >100 kb. Then, they further looked for replication in the German MI family study which involved 875 MI cases and 1644 controls using the GeneChip Human Mapping 500K Array Set (Affymetrix) [32]. The Same locus on chromosome 9p21 (rs1333049) had the strongest association with CAD in both studies with the risk increased by 36% per copy of the C allele. Of the nine loci which were shown to be strongly associated with CAD, two of these loci were able to replicate in the German MI study: chromosome 6q25.1 (rs6922269) and chromosome 2q36.3 (rs2943634). Further, the combined analysis of the two studies revealed four additional loci significantly associated with CAD: chromosome 1p13.3 (rs599839), 1q41 (rs17465637), 10q11.21 (rs501120) and 15q22.33 (rs17228212). In 2009, they performed another replication study with 11550 cases with CAD and 11205 controls from 9 European studies [33]. Other than the 9p21 locus, they confirmed significant association at 1p13.3 (rs599839), 1q41 (rs3008621) and 10q11.21 (rs501120). They were not able to show the significant association with 6q25.1 and 2q36.3 and there was no evidence for association with the locus at 15q22.33. The four loci (9p21, 1p13.3, 1q41 and 10q11.21) act independently and cumulatively increased the risk for CAD by 15% per additional risk allele. The genes located within or adjacent to the these four loci are listed in Table 4. The locus at chromosome 1p13.3 has been shown to be associated with increased plasma LDL cholesterol, and thus may contribute to CAD development [34–37]. 

The locus at 10q11.21 lies adjacent to the chemokine (C-X-C motif) ligand 12 gene (*CXCL12*) which encodes stromal cell-derived factor-1, a chemokine which plays a important role in stem-cell homing and regeneration of myocardial tissue in ischemic cardiomypopathy [38] and in promoting angiogenesis by recruiting endothelial progenitor cells from the bone marrow [39]. The SNPs at 1q41 locates within the melanoma inhibitory activity family, member 3 (*MIA3*) gene [40]. Underlying mechanism how these genetic loci affect the pathogenesis of CAD need to be further investigated. 

The meta-analysis of aforementioned three studies [24, 27, 32] and 7 additional case-control studies successfully replicated the significant association between the risk allele (C) of the lead SNP, rs1333049 at chromosome 9p21 and risk of CAD (OR= 1.29) [41]. These study have analyzed primarily on European descent, however, since the allele frequency differs among the different ethnic groups, the risk of CAD related to the SNPs at 9p21 may differ among each ethnic group. Since then, the replicated results in other ethnics such as Chinese, Japanese and Pakistanis are published and the association of the SNPs at 9p21 with CAD seems to be consistent among various ethic groups [42–45]. 

It is noteworthy that the SNPs in 9p21 region are also found to be associated with variety of diseases such as ischemic stroke (OR = 1.01–1.21) [46, 47], abdominal aortic aneurysm (OR = 1.31), intracranial aneurysm (OR = 1.29) [48], peripheral artery disease (OR = 1.29) [49], incident heart failure (OR = 1.17) [50], perioperative myocardial injury after coronary artery bypass graft surgery [51], type 2 diabetes (OR = 1.20) [52, 53].

More recently, the loci other than 9p21 have been shown to be associated with CAD or MI. Erdmann et al. applied less stringent statistical thresholds on their GWAS for CAD to identify any dismissed SNPs with modest effects or low allele frequencies and they found one new locus on 3q22.3 in muscle RAS oncogene homolog (*MRAS*) (OR = 1.15), the gene thought to play an important role in inflammation [54, 55]. 

There is another GWAS which identified the SNPs in the gene related to inflammation to be associated with MI [56]. They found five SNPs which affect eosinophil counts in blood in Icelandic population and reported that a nonsynonymous SNP at 12q24 in SH2B adaptor protein gene (3*SH2B3*) was associated with MI significantly (OR = 1.13). 

The GWAS of early-onset MI revealed 9 loci which have significant association [57]. Three of them were newly identified in the study: (i) an intergenic region between *MRPS6 *(mitochondrial ribosomal protein S6),* SLC5A3 *(solute carrier family 5 (sodium/myo-inositol cotransporter), member 3) and *KCNE2 *(potassium voltage-gated channel, Isk-related family, member 2) on chromosome 21q22 (OR = 1.19), 6p24 in* PHACTR1 *(phosphatase and actin regulator 1) (OR = 1.13) and 2q33 in *WDR12 *(WD repeat domain 12) (OR = 1.17). The mechanism by which genes at these three regions increases the risk of MI needs to be elucidated. In addition to the common variant, copy number variations can be analyzed by SNP chip and there were no common or rare copy number variations associated with risk of early-onset MI in this study. 

The group from the WTCCC/German MI study further conducted a genome-wide haplotype association study for the first time and identified the *SLC22A3-LPAL2-LPA* gene cluster on 6q26-27 as a strong susceptibility locus for CAD (OR = 1.8) [58]. An increased level of Lp(a) lipoprotein is a classical hereditary risk for CAD. Clarke et al. identified three chromosomal regions (6q26-27, 9p21 and 1p13) were strongly associated with the risk of CAD using the Human CVD Bead Chip which included 48742 markers relevant to cardiovascular disease on 6500 subjects. Among them, the *LPA *locus on 6q26-27 encoding Lp(a) lipoprotein had the strongest association. They identified two *LPA* variants that were strongly associated with both an increased level of Lp(a) lipoprotein and an increased risk of CAD (OR = 1.70 and 1.92) [59]. Both variants were strongly associated with a reduced copy number in *LPA* kringle IV-type 2 repeats and an increased level of Lp(a) lipoprotein. After adjustment for the Lp(a) lipoprotein level, the association between the *LPA *genotype score and the risk of CAD was abolished. The importance of classical risk factor Lp(a) was reemphasized by the GWAS.

## 4. Limitation of GWAS

Initially, GWAS have been primarily assessed only on European descent and the results of these GWAS may not be applicable to other ethnics due to wide difference of distribution of SNPs and allele frequency. Further studies for various ethnicity need to be done with use of newer chips which contains 1 million SNPs to increase coverage.

The CAD associated loci have been found in regions without known gene-encoding loci. Therefore, further studies will be required to elucidate the exact functional mechanism by which these loci modulate CAD risk.

Utility of genotyping 9p21 for clinical risk assessment is controversial [60–62]. The odds ratios for CAD risk in each selected SNPs are small (around 1.2) and explain only a small proportion of the heritable, genetic component of susceptibility to the disease. Newer susceptibility loci for CAD need to be validated with replication studies and in the future, we should evaluate the genetic risks by combining multiple independent common variants susceptible for CAD. 

The GWAS method is supported by the common disease-common variant hypothesis, which predicts that genetic variants causing common disease exist frequently, but each variant only have a small effect on disease susceptibility. Another hypothesis is the rare variant hypothesis. Rare variants have a minor allele frequency of less than 1%. The rare variant hypothesis postulates that common disease is caused by multiple rare variants which have a strong causative effect on disease and this hypothesis was confirmed in colorectal adenomas [63, 64]. Rare variants cannot be captured by GWAS and requires whole genome sequencing using next generation sequencing system. 

In addition, other types of variants, such as insertion-deletion variant, block substitution and inversion variant, so called structural variants may account for important contributors to the diseases and are also hard to detect by the chip-based method. The next generation sequencing method is also helpful to find these structural variants. 

## 5. Conclusion

(1) The SNPs Data from the HapMap project and development of new chip technology enabled genotyping large amount of common variants simultaneously and contributed to efficiently identify gene loci affecting susceptibility to common diseases including CAD.

(2) The region at 9p21 was shown to be significantly associated with CAD in 2007 and comprehensive replication across multiple studies provides unequivocal evidence that this locus is associated with CAD in European descent. This region is also associated with abdominal aneurysm, intracranial aneurysm and type 2 diabetes, and seems to be a very important region for various diseases.

(3) Since the odds ratios of the risk allele at 9p21 for CAD are small, screening for this risk allele probably affects little, if any, to the each individual's risk prediction. Using genomic tests to improve existing risk models would likely require combining the effects of multiple common genetic variants.

(4) Rare variants and structural variants which cannot be captured by GWAS need to be searched by whole genome sequencing.

(5) GWAS approach has identified novel and unbiased genetic contributors to CAD and these insights provide the important direction to better understand the pathogenesis of CAD and to develop new and improved preventive measures and treatments for CAD.

## Figures and Tables

**Table 1:** The well-known genetic polymorphisms which are thought to be associated with myocardial infarction or coronary artery disease.

Location	Gene name/Polymorphisms
17q23	Angiotensin-Converting Enzyme insertion/deletion (intron 16)
1q42-q43	Angiotensinogen Met235Thr, −6G/A
3q21-q25	Angiotensin II type1 Receptor 1166A/C
8q21-q22	Aldosterone Synthase (CYP11B2) −344T/C, Lys173Arg
14q32.1-q32.2	Bradykinin B2 receptor gene −58T/C
6p24.1	Endothelin-1 Lys198Asn
7q36	eNOS Glu298Asp, −786T/C
17q21.32	Glycoprotein IIIa P1A1/A2
5q23-31	Glycoprotein Ia 807T/C
17pter-p12	Glycoprotein Ib*α* Thr145Met
4q28	*β*fibrinogen −455G/A
11p11-q12	Prothrombin 20210G/A
7q21.3-q22	PAI-1 4G/5G (promoter region)
7q21.3	Paraoxonase1 Arg192Gln, Leu54Met
8p12-p11.2	Werner Helicase Gene Cys1367Arg
1p36.3	Methylenetetrahydrofolate reductase 677C/T
16q24	NADH/NADPH oxidase p22phox 242C/T, 640A/G
5q31.1	CD14 Monocyte Receptor −260C/T
11q22.3	Stromelysin (MMP3) 5A/6A (promoter region)
20q11.2-q13.1	Gelatinase B (MMP9) −1562C/T
19q13.2	ApolipoproteinE E2/E3/E4
16q21	Cholesteryl Ester Transfer Protein (CETP) Ile405Val
9q31.1	ABCA1 gene Ile823Met
3p25	PPAR-gamma Pro12Ala, Pro115Gln
20q13.11-q13.13	Prostacyclin synthase gene
The number of 9-bp (CCGCCAGCC) repeats (promoter region)	
17q11.2-q21.1	MCP-1 −2518G/A

**Table 2:** Representative loci associated with CAD identified in the family-based studies.

Reporter (year)	Race	locus	LOD score	causative gene
Pajukanta et al. [10]	Finland	2q21-22	3.7	no gene identified
		Xq23-26	3.5	no gene identified
Wang et al. [11]	USA	15q26	4.19	MEF2
Helgadottir et al. [12]	Iceland	13q12-13	2.86	ALOX5AP
Helgadottir et al. [13]	Iceland	17q22	NA	LTA4H

NA = not available.

**Table 3:** Representatives of chip-based GWAS of CAD/MI.

Author, year	Phenotype	No. of cases/controls	Chromosomal loci	OR
Helgadottir et al. [24]	MI	4587/12767	9p21	1.28
McPherson et al. [27]	CAD	3505/18745	9p21	1.26 (CCHS study)
			9p21	1.16 (CCHS study)
WTCCC [31]	CAD	2000/3000	9p21.3	1.47
Samani et al. [32]	CAD	2801/4582	9p21.3	1.28 (adjusted German)
			6q25.1	1.23 (adjusted German)
			2q36.3	1.08 (adjusted German)
			1p13.3	1.29
			1q41	1.2
			10q11.21	1.33
			15q22.33	1.21
CAD Consortium, [33]	CAD	11550/11205	9p21	1.2
			1p13.3	1.13
			1p41	1.1
			10q11.21	1.11
Clarke et al. [59]	CAD	7991/7946	6q26-27	1.51

**Table 4:** Genes located within or adjacent to the loci associated with CAD/MI.

Chromosome loci	Genes
1p33	PSRC1, CELRS2, MYBPHL, SORT1
1q41	MIA3
2q33	WDR12
2q36.3	no recognized genes
3q22.3	MRAS
6p24	PHACTR1
6q25.1	MTHFD1L
6q26-27	LPA
9p21	p16/CDKN2A, p15/CDKN2B, p14/ARF, MTAP, ANRIL
10q11.21	CXCL12
12q24	3SH2B3
21q22	MRPS6, SLC5A3, KCNE2
